# Supplementary material for: Effects of peer tutoring on middle school students’ mathematics self-concepts
Source: PLoS One. 2020 Apr 10;15(4):e0231410. doi: 10.1371/journal.pone.0231410 (PMC7147781; doi:10.1371/journal.pone.0231410)
Supplement: Attachment — Submitted filename: response_to_editor.docx [file pone.0231410.s001.docx]

PONE-D-19-28784R1
Effects of peer tutoring on middle school students’ mathematics self-concepts
PLOS ONE

Dear Editor, 

Thank you very much for all the work you are putting in to improve our manuscript. As you indicated us in the email, we added the following statement at the beginning of the Methods section "The institutional review board that authorized this research was the Valencian Ministry of Education. They approved the research but the obtained consent specified that data had to be analyzed anonymously".

As you suggested, we also indicated the type of consent the parents or guardians of the minors provided (written and informed consent). The Ethics Committee of the Spanish National Research Council (CSIC) did not specifically approve this study, so we did not indicate this information in the article.

If we must do any additional changes or we must address any other issues, please do not hesitate to contact us,

Thank you very much,

Kind regards,

The authors

**Academic Editor requirements**

When submitting your revision, we need you to address these additional requirements:

1. Please ensure that your manuscript meets PLOS ONE's style requirements, including those for file naming. The PLOS ONE style templates can be found at <http://www.plosone.org/attachments/PLOSOne_formatting_sample_main_body.pdf> and <http://www.plosone.org/attachments/PLOSOne_formatting_sample_title_authors_affiliations.pdf>

We ensured that PLOS ONE’s style requirements were met, including those for file naming

2. You indicated that you had ethical approval for your study. In your Methods section, please ensure you have also stated whether you obtained consent from parents or guardians of the minors included in the study (your manuscript only mentions participants' "families") or whether the research ethics committee or IRB specifically waived the need for their consent.

Now this part is clearly stated in the Methodology section (sample access subsection)

3. Your ethics statement must appear in the Methods section of your manuscript. If your ethics statement is written in any section besides the Methods, please move it to the Methods section and delete it from any other section. Please also ensure that your ethics statement is included in your manuscript, as the ethics section of your online submission will not be published alongside your manuscript.

- In the Methodology section we indicated that Ethical requirements provided by the Ethics Committee of the Spanish National Research Council (CSIC) were met during this research.


**Reviewer #1 comments:**

This manuscript reported an empirical study that was conducted in a Spanish middle school with the purpose of investigating the effects of peer tutoring on students’ math self-concept. Considering the school context, the effect sizes reported in the manuscript are not small and I believe may have practical significance. However, the study has one major flaw, which is not having a student achievement related variable. The only variable that the authors looked at was math self-concept. In addition, the statistical analysis reported in the results section were not clearly written and may be redundant.

You are absolutely right about the absence of a student achievement related variable. We, the authors, thought about this possibility before starting the research. Nevertheless, we found several legal problems when accessing students’ academic achievement in mathematics. Legal consent for the self-concept variable was not very difficult to obtain, but that was not the case for students’ academic achievement. The administration was reluctant to authorize us to use students’ academic information. We take note of your comment and we hope that we can incorporate this variable in our future studies somehow.

Detailed comments are listed below.
Major issues

1. Some important articles are missing from the literature review part. For example, Roscoe and Chi (2007) “Understanding Tutor Learning: Knowledge-Building and Knowledge-Telling in Peer Tutors’ Explanations and Questions”, Roscoe and Chi (2007) “Tutor learning: the role of explaining and responding to questions”.

Both references and other recent articles were added in the literature review part.

2. As the authors stated in the self-concept section, self-concept can influence academic achievement. So why was this not accounted for in the hypothesis formulation?

As we indicated before, we did not obtain legal consent to use the academic achievement variable in our research. Although we knew that we could not perform an analysis, we wanted to note the importance of self-concept regarding mathematics achievement. If you think we must delete this part, please, just tell us and we will do it.

3. What are the potential causes of research design that affect the results? If random assignment to conditions was implemented successfully, I don’t see any problems for a pretest-posttest, experimental vs. control group design.

Our apologies for the misunderstanding. We clarified this part. What Stigmar (2016) means is that some research designs such as pretest posttest without control group or posttest only with control may overestimate the effects of a peer tutoring experience. That’s why we wanted to note that it was important to select a pretest posttest with control group design, so that reported effects were as realistic as possible.

4. Please be specific about how students were assigned to different conditions. What did the authors mean by a probabilistic base?

We clarified this part. We indicated that half the students in each course were randomly assigned to the experimental group and the other half to the control group. There were six subgroups in each grade. A draw was performed for each grade so that three subgroups were assigned to experimental conditions and the other three to control conditions. An additional final draw was performed in some subgroups to exclude some students so that the number of students in the experimental group matched the number of students in the control group in each grade. 9 students were randomly excluded due to this procedure.

5. What were the subscales for the Marsh self-concept questionnaire? I suggest providing some sample items. Also, were there reversely coded items?

The three subscales were indicated. We also indicated the existence of reversely coded items in the questionnaire and provided some sample items.

6. Did the teacher do the lecture or provide more problems for students to solve in the control condition?

We now have indicated in the subsection “organization and scheduling” that students The same teacher that did the lecture in the experimental group also did it in the control group for the same grade. Students in the experimental and control groups were provided the same problems and exercises during the development of the peer tutoring intervention.

7. Please provide more details about the peer tutoring training that students received.

Further details regarding the peer tutoring training were provided in the “organization and scheduling” section.

8. What if both students who were paired had mistakes in their solved problems? Did they receive help from the teacher until they knew how to correctly solve the problem?

Our apologies for not including this information in the manuscript. Of course, if both students who are paired had mistakes in their solved problems, the teacher had to help them until they knew how to correctly solve the problem. This is now clearly stated in the “organization and scheduling” section.

9. Which group was the experimental group? Group A or group B?
10. Line 293, what did the three tests (test 1, 2 and 3) about?
11. I cannot understand Table 3.

Our apologies for not clarifying enough what group A and group B meant and the analysis we were carrying out in table 3. We have clarified this in the manuscript right now before table 3.

First, analysis of differences for the pretest scores between experimental and control groups were conducted for each grade (tests 1 to 3). Then, analysis of differences between the posttest and the pretest scores were conducted for the experimental group by grade and overall (tests 4 to 7) and, after that, analogous analysis were carried out for the control group (tests 8 to 11). Finally, a comparison of increments, that is, the difference between the posttest and the pretest scores for experimental and control group were carried out by grade and overall (tests 12 to 15). Group A and group B were just named like that so that the reader could understand that two subgroups or groups were being compared and which of them was the minuend and the subtrahend when calculating mean differences in table 3.

12. One major issue regarding the results is that the authors did not specify the independent variables and dependent variable in each statistical test they conducted.

The mathematics self-concept scores acts as the dependent variable and the different groups are the independent variables. This has been clearly stated in the manuscript.

13. Line 315. I suggest clearly stating what significant improvements were revealed.

This has been clearly stated.

14. I suggest restating the hypotheses in Discussion section.

Hypotheses were restated in Discussion section

15. I did not realize that the authors were also interested in the effects of different grade levels on self-concept until I finished reading the results and discussion. So I suggest including some literature in the literature review section.

Some literature regarding effect sizes in the literature review section has been included

Minor issues
16. It is not clear what global effect size means in the abstract.

“Global effect size” was changed by “overall effect size”. We just wanted to mean that this was the effect size for all grades combined and not separately.

17. First line on page 5, I think the word primary was missing from “…during the transition from school to…”

As, in all comments above, you are absolutely right. The word primary was added. Thank you very much.

18. The sentence from line 148 – 152 is too long and difficult to understand.

This sentence was divided in three separate sentences so that readers may understand it better. Thank you very much for your review.

**Reviewer #2 comments:**

Effects of Peer Tutoring On Middle School Students’ Mathematics Self-Concept
The Marsh questionnaire was used to measure participating students’ mathematics self-concepts before and after implementation of a peer-tutoring program.
The main aim of this research was to determine the effect of peer tutoring on middle school students’ mathematics self-concepts. The study tested two hypotheses.
Hypothesis 1: Students’ mathematics self-concepts will improve significantly as a result of peer tutoring.
Hypothesis 2: Students enrolled in different grades will not show significant differences regarding mathematics self-concepts before and after peer tutoring. (This hypothesis is not clear. I had to get to line 328 to get the point of this hypothesis. )

Our apologies for the lack of clarity regarding hypothesis 2. We have rewritten this hypothesis and we hope that now it looks clearer for the readers.

Relevant literature was used to discuss the impact of self-concept on the learning of mathematics. However, it would make the paper more meaningful if the authors could be more expansive how positive self-concept helps with mathematics performance.

In subsection “self-concept” we explained more how positive self-concepts helps with mathematics performance.

Line 101 the point that students experience important self-concept changes resulting in a lower mathematics self-concept. Line 101 is circuitous, as it does not explain why there should be changes now and what these changes could affect mathematics performance.

The information in this line has been rewritten so that it looks clearer for readers

In the section Peer tutoring and self-concept, it would be helpful if the authors could draw upon the literature cited to provide more substantial discussion how peer tutoring improve self-concept of the tutor.

More substantial discussion was provided from the cited literature in this subsection.

In line 144, the evidence is not as conclusive as one would hope. Perhaps the authors could offer reasons why the evidence are not so conclusive and how the current authors’ work could provide data to support the effect of peer-tutoring and tutors self-concept. More hypothesising here would make a better case for the hypothesis 1.

As in all comments above, you are absolutely right. More reasons on why the evidence are not so conclusive were given in this part and this made a better case for hypothesis 1. Thank you very much.

Hypothesis 2 is not clear. Perhaps the authors could provide clearer discussion and draw the reader to the meaning of this Hypothesis.

As stated above, hypothesis 2 was written.

The data collection process was discussed and easy to follow

Thank you very much for your kindness.

More discussion could be used to explain the formation of the tutor-tutee pair.

More discussion was used in this section.

Although the authors cited the literature (De Becker et al.) they drew upon to construct the tutor-tutee pair, what other methods did they consider to form the tutor-tutee pair and why the work of De Becker chosen, In particular how much difference in performance should there be between each member of the pair? Only when the relevant literature has been discussed could the authors use the literature to support their selection of the tutor-tutee pair.

This was also discussed in the section. The other main option instead of reciprocal peer tutoring was fixed peer tutoring. Fixed peer tutoring implies a different type of pairing than reciprocal peer tutoring. All this is discussed now in the manuscript.

Did the authors conduct a pilot study to help confirm such tutor-tutee pair would be effective? Without such preparation, it is difficult to give much credence to the effects of the study.

A pilot study was carried out one year before. Although only 19 students participated in the study, promising results were found. This study was published and it is referenced in the manuscript.

In the current paper, the authors explained the formation of the pairs. The students were arranged according to their performance, thus the first student was paired with the second, the third with the fourth, and so on. The authors, however, did not explain how different were the students in each pair. What if the first student was an A+ student (85 marks) and the second student, also an A+ student (83 marks), one would not pair these two students as the peer tutoring process would not be helpful to either one. This may explain the finding why although peer tutoring frequently has a positive impact on students,“there is usually a low percentage of students (about 10–15%) that do not improve academically or psychologically. This may be because some students do not like to help other peers with academic tasks, and, although the main goal of peer tutoring is to foster collaboration, the reluctance of some students is so strong that interactions are not valuable and learning between peers does not take place.” Line 338 -343. It could be that when the differences between the pairs are so limited, such pairs do not benefit from the experience. Did the authors interview such students to ascertain why they did not benefit from the experience?

You are absolutely right. We included your reflection about what happens when differences peers are so limited. Besides, ceiling effects were also indicated as some students already had high scores in the pretest. We did not interview students, but we take note of the importance of using qualitative information to contrast these findinds for our future research. Thank you very much for improving our manuscript.
