# Supplementary material for: Effects of peer tutoring on middle school students’ mathematics self-concepts
Source: PLoS One. 2020 Apr 10;15(4):e0231410. doi: 10.1371/journal.pone.0231410 (PMC7147781; doi:10.1371/journal.pone.0231410)
Supplement: Attachment — Submitted filename: response_to_reviewers.docx [file pone.0231410.s002.docx]

PONE-D-19-28784R1
Effects of peer tutoring on middle school students’ mathematics self-concepts
PLOS ONE

Dear Editor and Reviewer, 

Thank you very much for all the work you are putting in to improve our manuscript. As you suggested, we addressed the minor revisions of one of the reviewers and we hope that our manuscript is now suitable for publication. Our responses to these revisions may be found below.

If you think we must do any additional changes or we must address any other issues, please do not hesitate to contact us,

Thank you very much,

Kind regards,

The authors

**Reviewer #1 comments:**

I enjoyed reading this manuscript. I appreciate the authors’ effort in addressing the concerns I raised in the previous review and understand the difficulty in obtaining student achievement data. I hope the minor issues listed below could help the authors further improve their manuscript.

Thank you very much for your thorough reviews that are helping us so much to improve our manuscript.

1. Last sentence on page 4 may have typos. “…such as motivation oron…”

Our apologies for that. We accidentally deleted a space between “or” and “on” and it became a typo. This was corrected and now it says what it was intended to say “such as motivation **or** on task behavior”.

2. In the section Peer tutoring and self-concept, the authors first stated research related to peer tutoring in math on self-concept, then described related research in other fields. However, the authors stated research in math again at the end of this section. I suggest enhancing the structure of this section.

You are absolutely right. We restructured this section by dividing all the information that was on it into two different sections. The first one called “peer tutoring and self-concept” now addresses experiences in subjects different of mathematics. The second one called ““peer tutoring and mathematics self-concept” specifically addresses peer tutoring and mathematics self-concept experiences.

3. I did not see that the authors elaborated on the peer tutoring literature on how students in different grades, although the authors responded that they did. From my point of view, such elaboration can shadow Hypothesis 2.

Sorry for that. We included the following information at the end of the “peer tutoring” section. Academic and psychological effects of peer tutoring may differ significantly across educational levels. For example, academic effects are usually greater in primary education than in secondary education [47, 48]. Nevertheless, effects within the same educational level are expected to be similar and, when analyzing differences across grades, significant differences are rarely reported [49, 50].

4. The results section is not very readable. For example, what type of statistical tests were test 1 to test 3? I suggest the authors refer to APA style guide to get some ideas about how to write a results section.

We indicated that tests 1 to 3 refer to analysis of differences for pretest scores between experimental and control groups for each grade. We also looked for APA style guides to report results. APA guide indicates that inferential statistical tests, tables and figures must be included and that effect sizes must be reported. As there were no figures in our manuscript, we included two figures (figure 1. Experimental vs control group overall scores and figure 2. Experimental scores by grades) so that readers of the manuscript could understand better the results section.

5. I raised the issue in the previous review that the analysis the authors conducted were somewhat redundant, such as the ANOVA and the t tests. However, the authors did not address this in the revision.

In the first ANOVA we addressed the differences between the pretest scores for the experimental group across grades and in the second ANOVA we addressed the differences in the increments for the experimental group across grades. These analysis had not been previously performed in the previous t tests, that’s why we did not delete any of these analysis. However, if you think we must delete any of these analysis, please just tell us and we will do it.
