# Supplementary material for: Effects of peer tutoring on middle school students’ mathematics self-concepts
Source: PLoS One. 2020 Apr 10;15(4):e0231410. doi: 10.1371/journal.pone.0231410 (PMC7147781; doi:10.1371/journal.pone.0231410)
Supplement: Attachment — Submitted filename: response_to_editor_and_reviewers.docx [file pone.0231410.s004.docx]

PONE-D-19-28784R3
Effects of peer tutoring on middle school students’ mathematics self-concepts
PLOS ONE

Dear Editor, 

Thank you very much for all the work you are putting in to improve our manuscript. As you suggested, we addressed the issues related to data presentation and analysis. Our responses to the last revision may be found below.

If you think we must do any additional changes or we must address any other issues, please do not hesitate to contact us,

Thank you very much,

Kind regards,

The authors

**Editor comments:**

1. The titles of Tables and Figures need to be more explicit so as to reflect the content of Tables and Figures.

We included more information on the titles of tables and figures so that they could be more explicit.

2. There is still a lot of redundant information in the tables. For example, the pre-test and post-test in Table should appear only once rather than multiple times in the table (see Ngu & Phan, 2017, page 888).

We checked your article in *International Journal of Mathematical Education in Science and Technology* and carefully looked at how you presented the data in the tables. Pretest and posttest only appear once in each table.

3. What do you mean by students’ t-test? Researchers normally use a t-test to examine the mean difference between two groups. I never heard of a student’s t-test.

As you indicate, we just wanted to mean a t-test. We deleted all “students’ t-test” and replaced them by “t-test” in the manuscript.

4. The analysis of ANOVA is very confusing. I suggest you re-analyse the data. You can either perform a repeated measures ANOVA or t-test. You may perform a 2 (group: experimental vs. control) x 2 (test: pre-test vs. post-test) ANOVA for each grade separately and then for the all grades combined. The group is a between-group factor, and the test is a repeated measures within-subject factor. Alternatively, perform a t-test between the experimental group and the control group on gain score (i.e., post-test – pre-test) for each grade separately and then for all grades.

The t-test between the experimental group and control group on gain score was exactly what we meant by “increments” in our previous submission. We changed the word “increments” by “gain scores” as this analysis had been previously performed in the manuscript.

**Reviewer #1 comments:**

I enjoyed reading this manuscript. I appreciate the authors’ effort in addressing the concerns I raised in the previous review and understand the difficulty in obtaining student achievement data. I hope the minor issues listed below could help the authors further improve their manuscript.

Thank you very much for your thorough reviews that are helping us so much to improve our manuscript.

1. Last sentence on page 4 may have typos. “…such as motivation oron…”

Our apologies for that. We accidentally deleted a space between “or” and “on” and it became a typo. This was corrected and now it says what it was intended to say “such as motivation **or on** task behavior”.

2. In the section Peer tutoring and self-concept, the authors first stated research related to peer tutoring in math on self-concept, then described related research in other fields. However, the authors stated research in math again at the end of this section. I suggest enhancing the structure of this section.

You are absolutely right. We restructured this section by dividing all the information that was on it into two different sections. The first one called “peer tutoring and self-concept” now addresses experiences in subjects different of mathematics. The second one called ““peer tutoring and mathematics self-concept” specifically addresses peer tutoring and mathematics self-concept experiences.

3. I did not see that the authors elaborated on the peer tutoring literature on how students in different grades, although the authors responded that they did. From my point of view, such elaboration can shadow Hypothesis 2.

Sorry for that. We included the following information at the end of the “peer tutoring” section. Academic and psychological effects of peer tutoring may differ significantly across educational levels. For example, academic effects are usually greater in primary education than in secondary education [47, 48]. Nevertheless, effects within the same educational level are expected to be similar and, when analyzing differences across grades, significant differences are rarely reported [49, 50].

4. The results section is not very readable. For example, what type of statistical tests were test 1 to test 3? I suggest the authors refer to APA style guide to get some ideas about how to write a results section.

Following suggestions given by the editor, tests 1 to 3 were deleted as they were not necessary. We also looked for APA style guides to report results. APA guide indicates that inferential statistical tests, tables and figures must be included and that effect sizes must be reported. As there were no figures in our manuscript, we included two figures (figure 1. Experimental vs control group overall scores and figure 2. Experimental scores by grades) so that readers of the manuscript could understand better the results section.

5. I raised the issue in the previous review that the analysis the authors conducted were somewhat redundant, such as the ANOVA and the t tests. However, the authors did not address this in the revision.

The editor raised the same issue as you did. Following your recommendations, we remade the entire results section, following APA style, trying to make it more readable and deleting redundant analysis. We hope that this section looks much better now.
